# Population-level impact of switching to 1-dose human papillomavirus vaccination in high-income countries: examining uncertainties using mathematical modeling

**DOI:** 10.1093/jncimonographs/lgae038

**Published:** 2024-11-12

**Authors:** Marc Brisson, Jean-François Laprise, Mélanie Drolet, Éléonore Chamberland, Élodie Bénard, Emily A Burger, Mark Jit, Jane J Kim, Lauri E Markowitz, Chantal Sauvageau, Stephen Sy

**Affiliations:** Département de Médecine Sociale et Préventive, Université Laval, Québec, Québec, Canada; Centre de Recherche du CHU de Québec - Université Laval, Québec, Québec, Canada; Centre de Recherche du CHU de Québec - Université Laval, Québec, Québec, Canada; Centre de Recherche du CHU de Québec - Université Laval, Québec, Québec, Canada; Centre de Recherche du CHU de Québec - Université Laval, Québec, Québec, Canada; Département de Médecine Sociale et Préventive, Université Laval, Québec, Québec, Canada; Centre de Recherche du CHU de Québec - Université Laval, Québec, Québec, Canada; Center for Health Decision Science, Department of Health Policy and Management, Harvard T.H. Chan School of Public Health, Boston, MA, USA; Department of Health Management and Health Economics, University of Oslo, Oslo, Norway; Centre for Mathematical Modelling of Infectious Disease, London School of Hygiene and Tropical Medicine, London, UK; School of Public Health, University of Hong Kong, Hong Kong SAR, China; Center for Health Decision Science, Department of Health Policy and Management, Harvard T.H. Chan School of Public Health, Boston, MA, USA; National Center for Immunization and Respiratory Diseases, Centers for Disease Control and Prevention, Atlanta, GA, USA; Département de Médecine Sociale et Préventive, Université Laval, Québec, Québec, Canada; Centre de Recherche du CHU de Québec - Université Laval, Québec, Québec, Canada; Institut National de Santé Publique du Québec et Direction Régionale de Santé Publique de la Capitale-Nationale, Québec, Québec, Canada; Center for Health Decision Science, Department of Health Policy and Management, Harvard T.H. Chan School of Public Health, Boston, MA, USA

## Abstract

**Background:**

A concern in high-income countries is that switching to 1-dose human papillomavirus (HPV) vaccination could cause a rebound in HPV infection and cervical cancer if 1-dose efficacy or duration were inferior to 2 doses. Using mathematical modeling and up-to-date trial-based data, we projected the population-level effectiveness of switching from 2-dose to 1-dose vaccination under different vaccine efficacy and duration assumptions in high-income countries.

**Methods:**

We used HPV-ADVISE (Agent-based Dynamic model for VaccInation and Screening Evaluation), a transmission-dynamic model of HPV infection and cervical cancer, varying key model assumptions to identify those with the greatest impact on projections of HPV-16 and cervical cancer incidence over time: 1) 1-dose vaccine efficacy and vaccine duration, 2) mechanisms of vaccine efficacy and duration over time, 3) midadult (>30 years of age) sexual behavior, 4) progression to cervical cancer among midadults, and 5) vaccination coverage and programs.

**Results:**

In high-income countries, 1-dose vaccination would cause no appreciable rebound in HPV-16 infection, except for a limited rebound under the most pessimistic assumptions of vaccine duration (average, 25 years), because 1) the switch would occur when HPV prevalence is low because of high 2-dose vaccination coverage and 2) individuals would be protected during their peak ages of sexual activity (<35 to 40 years of age). Our model projects a more limited rebound in cervical cancer because of a shift to older age at infection, resulting in fewer life-years left to potentially develop cancer. Projections were robust when varying key model assumptions.

**Conclusions:**

High protection during peak ages of sexual activity in high-income countries would likely mitigate any potential rebounds in HPV infection and cervical cancer under the most pessimistic assumptions of 1-dose efficacy and duration.

In 2022, based on evidence from clinical trials indicating high and sustained 1-dose protection against human papillomavirus (HPV) infection (1-3), the World Health Organization Strategic Advisory Group of Experts on Immunization announced that vaccination on a 1-dose schedule could be considered for individuals aged 9 to 20 years (4). The World Health Organization announcement was also based on model projections for low- and middle-income countries (LMICs), suggesting that 1-dose vaccination could avert a similar number of cervical cancers as 2 doses and would be a more efficient use of vaccine doses, if the average duration of protection from 1 dose is greater than 20 to 30 years (5,6). Since the announcement, 1-dose trial-based evidence has accrued: 1) Updated data from the Costa Rica Vaccine Trial (CVT) show stable antibodies up to 16 years for 1-dose vaccination (7,8); 2) updated data from the India cohort study show 92% vaccine efficacy against persistent HPV-16/18 infection, with a median 12 years of follow-up (9,10); and 3) the final results from the KENya Single-dose HPV-vaccine Efficacy (KEN SHE) randomized controlled trial have shown 98% efficacy for 1 dose after 3 years of follow-up (11).

Several high-income countries have adopted 1-dose HPV vaccination since the World Health Organization announcement (eg, Ireland, the United Kingdom, Australia), while others have decided to remain with 2-dose schedules (eg, the Netherlands, Sweden, Spain) or are examining the question (eg, the United States, Canada) (12,13). Most high-income countries have been vaccinating against HPV for more than a decade, with high coverage (14). A large proportion of these countries have a gender-neutral approach (>40%) and introduced multiage cohort vaccination (>80%) (14). These programs have led to substantial decreases in HPV prevalence and related diseases (15). For example, vaccine type-specific HPV prevalence has declined by about 90% in the United Kingdom and cervical intraepithelial neoplasia, grade 2 or worse by more than 70% in vaccinated age groups in the United States and United Kingdom (16-18). The main source of concern for policy makers in high-income countries is that switching to a 1-dose vaccination strategy could lead to a rebound in HPV infection and cervical cancer and jeopardize elimination efforts if 1-dose vaccine efficacy or duration were inferior to 2 doses (19). Previous modeling analyses for LMICs have suggested that the duration of protection is the key determinant of 1-dose vs 2-dose population-level effectiveness (5,6). Many factors could potentially mitigate or exacerbate the impact of limited duration of protection, such as 1) whether waning of protection occurs constantly after vaccination or declines after the peak ages of sexual activity and 2) the number of new sexual partners and rate of progression from infection to cervical cancer among older adults. A secondary source of concern in high-income countries is that there is less evidence of 1-dose vaccine efficacy for male than for female individuals and the potential impact that lower 1-dose efficacy among boys and men could have on the population-level effectiveness of 1-dose gender-neutral vaccination. Finally, it is unclear to what extent decades of high vaccination coverage and low HPV prevalence in high-income countries can offer resilience to any potential loss of vaccine efficacy or duration of protection following a switch to 1-dose vaccination by delaying or attenuating rebounds in HPV infection and cervical cancer.

Mathematical modeling has been a proven tool to inform previous HPV vaccination public health decisions by making projections under different “what-if” scenarios to illustrate uncertainty (eg, switch from 3 to 2 doses) (20-22). The objective of this study was to use mathematical modeling and up-to-date trial-based efficacy and durability data to examine the following key 1-dose public health questions in high-income countries: 1) What would be the population-level impact of switching from 2-dose to 1-dose vaccination if vaccine efficacy or duration of protection were inferior to 2 doses, and 2) under what conditions would a rebound in HPV and cervical cancer incidence occur following a switch to 1-dose HPV vaccination? To answer these questions, we used mathematical modeling to examine whether the following remaining uncertainties can have an impact on the population-level effectiveness of 1-dose vaccination for different high-income country settings: 1-dose vaccine efficacy and duration among female and male individuals, potential mechanisms of waning protection over time, and sexual activity and progression rates to cervical cancer among older adults.

## Methods

### Dynamic model descriptions

For model simulations, we used HPV-ADVISE (Agent-based Dynamic model for VaccInation and Screening Evaluation) (23-26). HPV-ADVISE is an individual-based transmission-dynamic model of partnership formation and dissolution as well as natural history of HPV infection and disease (see https://marc-brisson.net/HPVadvise-Can.pdf, https://www.marc-brisson.net/HPVadvise-US.pdf) for an in-depth description of the model). Individuals enter the modeled population before sexual debut and are assigned different risk factors for HPV infection and disease (4 sexual activity levels and 5 cervical screening behavior levels). HPV transmission is assumed to depend on sexual activity, probability of transmission, infectious period, and natural immunity. Partnership formation and duration are based on specific sex, age, and level of sexual activity partner acquisition and separation rates as well as mixing patterns. Eighteen HPV types are modeled individually. The natural history of cervical cancer is represented by HPV infection states (susceptible, infected, immune), 3 precancerous cervical lesion grades (cervical intraepithelial neoplasia, grades 1 to 3), and 3 cervical cancer stages. Transition rates between states are age and HPV type specific. The model can capture a wide variety of complex HPV vaccination and cervical screening strategies. For model projections, we used HPV-ADVISE US and HPV-ADVISE Canada, which were calibrated to highly stratified sexual behavior, HPV epidemiology, and cervical screening data from each country (for a total of >750 data points for each country). HPV-ADVISE models have been extensively peer reviewed and used to inform vaccination policy decisions in the United States, Canada, the United Kingdom, and globally (4,27-33). Furthermore, they have been validated against postvaccination data and other models (26,34-36). We used both the US and Canada HPV-ADVISE models to capture variability in sexual behavior and epidemiology between these countries and for generalization of results to other high-income countries.

### Vaccine efficacy and duration of protection

**Figure 1.**
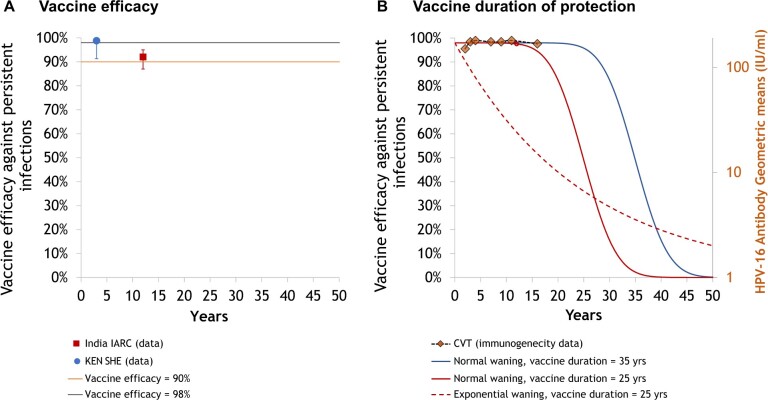
Vaccine efficacy and duration of protection data and model scenarios. **A)** Noninferiority scenario (vaccine efficacy = 98%, vaccine duration = lifelong) and pessimistic 1-dose vaccine efficacy scenario (vaccine efficacy = 90%) compared with vaccine efficacy against persistent HPV-16/18 infections among women in the IARC India study (10) and the KEN SHE trial (11). **B)** Pessimistic 1-dose average duration of vaccine protection scenarios, with a normal distribution (vaccine efficacy = 98%, vaccine duration scenario 1 = 25 years and vaccine duration scenario 2 = 35 years [standard deviation = 5 years]) and an exponential waning distribution (vaccine efficacy = 98%, vaccine duration = 25 years) compared with HPV-16 antibody geometric mean concentration in the CVT Trial (8). Of note, we assumed that vaccine efficacy functions as a take, meaning that in the base case, 98% of individuals are 100% protected per sex act after vaccination and 2% are 0% protected per sex act. In the waning scenarios (panel B), the proportion of individuals with 0% protection increases over time. CVT = Costa Rica Vaccine Trial; HPV = human papillomavirus; IARC = International Agency for Research on Cancer; KEN SHE = KENya Single-dose HPV-vaccine Efficacy.

The main source of concern regarding a switch from a 2-dose to a 1-dose HPV vaccination program is that 1 dose would have lower vaccine efficacy or shorter duration of protection than 2 doses. To explore this issue, we kept the 2-dose vaccine efficacy and duration constant and varied 1-dose characteristics, noting that there are also no long-term data for 2- or 3-dose protection. We assumed that 2-dose vaccine efficacy was 98% against nonavalent HPV-type infection and that duration of protection was lifelong. Different 1-dose vaccine efficacy and vaccine duration scenarios were modeled to explore their impact on projections (Figure 1):

- ***Noninferiority scenario (vaccine efficacy = 98%, vaccine duration = lifelong).*** The noninferiority scenario is based on the results from the International Agency for Research on Cancer (IARC) India study, the CVT, and the KEN SHE randomized trial, suggesting similar high and sustained protection for 1 and 2 doses (1-3,8,10,11). We chose 98% vaccine efficacy based on the final KEN SHE trial results (11). We assumed that 1-dose characteristics are the same for girls and boys.
- ***Pessimistic 1-dose vaccine efficacy scenarios (vaccine efficacy pessimistic scenario 1 = 90% for both genders, vaccine efficacy pessimistic scenario 2 = noninferior for girls and 70% for boys).*** We modeled 2 pessimistic 1-dose vaccine efficacy scenarios. In the first pessimistic scenario, we assumed 90% vaccine efficacy based on the lower bound of the 1-dose vaccine efficacy 95% confidence interval in the KEN SHE trial (11). In the second pessimistic scenario, given the greater uncertainty about 1-dose vaccine efficacy among boys, we assumed that vaccine efficacy was 70% for boys (keeping 1 dose noninferior for girls). For both scenarios, 1-dose duration of protection was assumed to be lifelong.
- ***Pessimistic 1-dose average duration of protection scenarios (vaccine duration pessimistic scenario 1 = 25 years, vaccine duration pessimistic scenario 2 = 35 years).*** We chose 25 and 35 years of average duration of protection for the pessimistic scenarios of vaccine duration because there is currently no evidence of 1-dose waning of protection after more than 12 and 16 years of follow-up in the IARC India study and the CVT, respectively (2,3,8,10). We modeled duration of protection using a normal distribution, with a 5-year standard deviation. Hence, for the most pessimistic assumption of an average 25 years of 1-dose protection, protection drops rapidly 15 years after vaccination, and almost all vaccinated individuals have 0% protection 35 years after vaccination (Figure 1).

### Vaccination program and coverage

In our base case, we modeled a vaccination program and coverage representing high-income countries with high gender-neutral HPV vaccination coverage, such as the United States, Canada, the United Kingdom, and Australia (14). In these high-income countries, girls-only bivalent or quadrivalent HPV vaccination was introduced between 2006 and 2008, followed by gender-neutral nonavalent vaccination between 2015 and 2021 (14,37-40). Currently, vaccination coverage with at least 1 dose is approximately 80% by 17 years of age in the United States (41), 85% among 9-year-old children in Québec, Canada (42), 85% among 10-year-old children in England (43), and more than 85% among 15-year-old adolescents in Australia (44). Based on these high-income countries, we modeled the following general scenario: introduction of quadrivalent 3-dose routine HPV vaccination of 10-year-old girls, with a catch-up in 2008 of individuals aged 11 to 19 years, and a switch to gender-neutral 2-dose nonavalent vaccination in 2016 (assuming that 2 doses are noninferior to 3 doses). We assumed that a switch to 1-dose vaccination would occur in 2024. Finally, we assumed that vaccination coverage was stable at 85% (ie, switching to a 1-dose schedule has no impact on vaccination coverage).

### Sensitivity analysis

We varied additional key model assumptions to identify and explain those that have the greatest impact on 1-dose vaccination projections: 1) mechanism of waning vaccine protection over time, 2) sexual activity of midadults, 3) progression to cervical cancer among midadults, and 4) vaccination coverage and vaccination programs:

- ***Mechanism of waning protection over time.*** There is uncertainty about how HPV vaccine protection could wane in the future because currently, there is no evidence of waning to infer decline functions (2,3,8,10). We modeled initial vaccine efficacy as a take per person (eg, 98% of individuals have 100% protection until they lose their protection). In our base case scenarios, we used a worst-case scenario, where protection immediately declines from 100% to 0% per sex act when protection wanes. In the sensitivity analysis, we examined an intermediate scenario where protection declines from 100% to 50% per sex act when protection wanes. In addition, we examined the impact of an exponential waning function, where protection starts declining immediately after vaccination. Although an exponential decline in protection is not consistent with current empirical data (Figure 1), we included the scenario because it has been used in prior modeling studies (19) and to illustrate the importance of vaccine protection during the peak ages of sexual activity.
- ***Sexual activity among midadults*.** Although there are population-based estimates of the lifetime number of sexual partners in high-income countries by age and gender, data are scarce on the number of new sexual partners by age, except for a few studies, such as the National Survey of Sexual Attitudes and Lifestyles (45). Furthermore, data on the lifetime number of sexual partners by age are different for male and female individuals and are affected by age-period-cohort effects (eg, female individuals have a declining number of lifetime partners after 40 years of age) (46-49). Hence, there is uncertainty about the number of new partners among midadults, the point at which 1-dose protection could potentially wane. We examined lower and higher sexual activity scenarios by multiplying base case partner separation and acquisition rates among individuals older than 30 years of age by 0.5 and 1.5, respectively.
- ***Progression to cervical cancer among midadults*.** Because there are fewer incident HPV infections among women older than 30 years of age (50,51), there remains considerable uncertainty about the relative progression of incident HPV infection to cervical cancer among this age group compared with younger women (50-53). We examined faster and slower progression scenarios for women older than 30 years of age by multiplying progression rates between cervical intraepithelial neoplasia grade 3, and cervical cancer by 2 and 0.5, respectively.
- ***Vaccination coverage*.** Given the variability in vaccination programs and coverage between high-income countries (13,14), we examined the sensitivity of model projections when assuming a girls-only HPV vaccination program or lower vaccination coverage (65%). Our base case of 85% can represent a large number of high-income countries in Europe, North America, and Australia, such as the United States, Canada, the United Kingdom, Spain, Portugal, Ireland, Sweden, Denmark, Norway, Iceland, and Australia (range = 83%-96%) (13,41,43). Lower (65%) coverage may represent countries such as the Netherlands, Germany, Switzerland, Austria, France, Italy, Finland, and New Zealand as well as some Canadian provinces and US states (range = 50%-79%) (13).
- ***Between-model validation.*** We conducted between-model validation to assess the robustness of conclusions to model structure and parameter assumptions, as recommended (36,54). We ran the base case 1-dose vaccine efficacy and duration scenarios with the Harvard model. The Harvard model has also been extensively published (55-58) and validated against other models through the Cancer Intervention and Surveillance Modeling Network and the Cervical Cancer Elimination Modeling Consortium (59,60).

### Outcomes

We used 2 primary health outcomes: HPV-16 infection among female individuals and cervical cancer (squamous cell carcinoma). We chose HPV-16 infection because it is the type most associated with cervical cancer in high-income countries (61). HPV-16 is also estimated to have the highest force of infection and transmissibility (as measured by its reproduction number, R0) (62,63) and therefore is the type most difficult to control and would theoretically have the greatest rebound. We examined HPV-16 infection among male individuals and any HPV vaccine type as secondary outcomes.

### Statistical analysis

We projected 4 main model outcomes: the 1) relative change in HPV-16 infection incidence among female individuals over time vs no vaccination, 2) relative change in cervical cancer incidence over time vs no vaccination (cervical cancers attributable to all HPV types), 3) median age of HPV-16 infection, and 4) percentage change in the cumulative incidence of cervical cancer over a 100-year time horizon (2- or 1-dose vaccination vs no vaccination and 2-dose vs 1-dose vaccination). Variability of model projections is presented as the median, 10^th^, 25^th^, 75^th^, and 90^th^ percentiles (referred to as the Interquartile range (IQR) and 80% Uncertainty Interval (80% UI) of simulations) from 100 parameter sets (50 best fitting parameter sets for both HPV-ADVISE US [http://www.marc-brisson.net/HPVadvise-US.pdf] and HPV-ADVISE Canada [https://marc-brisson.net/HPVadvise-Can.pdf]). The uncertainty interval represents uncertainty about sexual behavior and biological parameters as well as variability in the epidemiological data used for model calibration.

## Results

### Population-level impact of switching to 1-dose vaccination

#### Two-dose and noninferior 1-dose vaccine efficacy and duration

**Figure 2.**
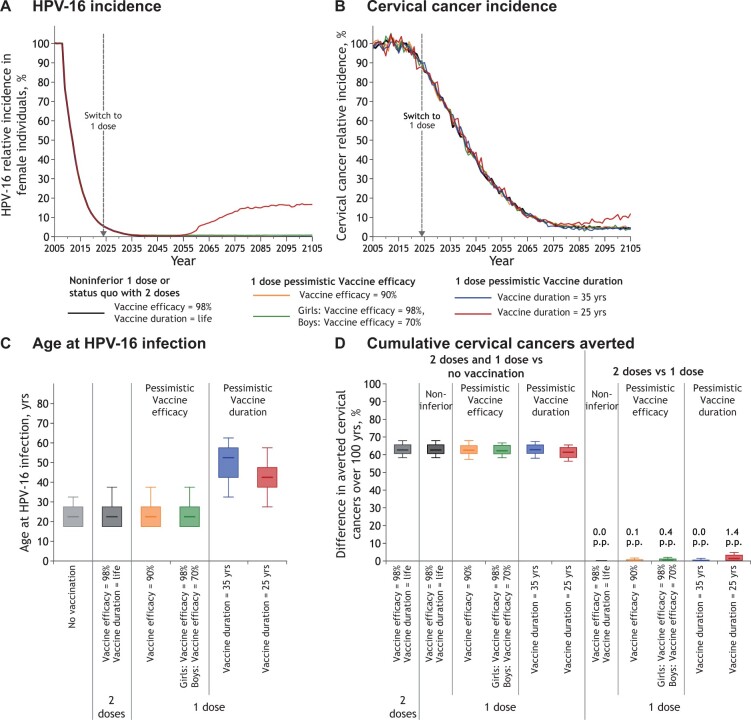
Projected population-level impact of switching to 1-dose HPV vaccination for different vaccine efficacy and vaccine duration of protection scenarios. Relative incidence of **A)** HPV-16 infection among female individuals and **B)** cervical cancer (vs no vaccination). The lines are the median result of model projections using 100 parameter sets. The lines overlap for all scenarios during the first years after the start of vaccination and over 100 years for all scenarios, except vaccine duration = 25 years. **C)** Age of HPV-16 acquisition among female individuals before vaccination (no vaccination) and at postvaccination equilibrium. Box plots represent the median, 10^th^, 25^th^, 75^th^, and 90^th^ percentiles of age at HPV-16 infection among the parameter sets that do not lead to elimination of HPV-16. **D)** Percentage change in the cumulative incidence of cervical cancer over 100 years (2-dose or 1-dose vaccination vs no vaccination and 2-dose vs 1-dose vaccination for different 1-dose scenarios). Box plots represent the median, 10^th^, 25^th^, 75^th^, and 90^th^ percentiles of model projections using 100 parameter sets. HPV = human papillomavirus; p.p. = percentage point difference.

The model projected that by 2045, 2-dose or noninferior 1-dose gender-neutral HPV vaccination will lead to near elimination of HPV-16 and reduce cervical cancer incidence by more than 50% in high-income countries with high coverage (Figure 2, A and B). Under these assumptions, HPV vaccination is projected to reduce cervical cancer incidence by more than 85% before the end of the century (Figure 2, B), averting about 60% of all cervical cancers (vs no vaccination) over 100 years (Figure 2, D).

#### One-dose vaccine efficacy and mitigating the impact of herd effects

Even under pessimistic scenarios of 90% vaccine efficacy for both genders or substantially inferior 1-dose 70% efficacy for boys, 1-dose vaccination is projected to produce similar population-level effectiveness against HPV infection and cervical cancer as 2 doses (projections overlap, Figure 2 for outcomes among female individuals and Supplementary Figure S2, A and B, available online, for male individuals). This result stems from the projected herd effects in highly vaccinated populations with gender-neutral vaccination which provides indirect protection for unvaccinated or unprotected vaccinated individuals, thus mitigating potential loss of vaccine efficacy with 1 dose.

#### One-dose duration of protection and mitigating the impact of shift in the age at infection

Under most assumptions, no rebound in HPV infection or cervical cancer is observed (Figure 2, A and B; Supplementary Figure S2, available online). Only under our most pessimistic assumption of duration of protection (vaccine duration =25 years) does the model project a rebound in HPV infection and cervical cancer incidence. Assuming a 25 year average duration of protection, the model projects that 1-dose vaccination would lead to a rebound in HPV-16 infection starting about 25 years after a switch to 1 dose, with an equilibrium incidence about 15 to 20 percentage points higher than with 2 doses (Figure 2, A; Supplementary Figure S2, A, available online). The rebound is projected to occur decades after a switch to 1-dose vaccination because 1) HPV-16 incidence is low at the time of the switch following 10 to 15 years of high HPV vaccination coverage (ie, >90% reduction vs prevaccination) and 2) time is needed for the first 1-dose cohorts to start losing protection (around 25 years after the switch to 1 dose, assuming vaccine duration =25 years). In addition, the rebound in HPV-16 infection is projected to be limited in the long term because 1) individuals are protected during their peak ages of sexual activity (ie, protected from sexual debut to 35 years of age, assuming vaccination at age 10 years and vaccine duration = 25 years) and 2) there are few expected new partners once full protection would wane (after age 35 years) (Supplementary Figure S1, available online). Hence, at the population level, high effective vaccination coverage among the younger, more sexually active age groups (ie, high percentage of the younger population vaccinated and protected) provides direct protection within these age groups and herd effects to older, unprotected age groups (due to waning) that have a lower average number of new sexual partners. When assuming an average duration of protection of 35 years, 1-dose vaccination is projected to produce similar population-level effectiveness against HPV infection as 2 doses (projections overlap, Figure 2, A and B; Supplementary Figure S2, available online); 35 years of protection with high gender-neutral vaccination coverage is thus projected to provide sufficient herd effects to mitigate losses in 1-dose protection later in life.

A switch to 1-dose vaccination is projected to lead to a more limited and delayed rebound in cervical cancer incidence than in HPV infection, when assuming pessimistic duration of protection (Figure 2). Assuming a 25 year average duration of protection, the model projects that 1-dose vaccination would lead to about a 5-percentage-point increase in cervical cancer incidence at equilibrium, starting more than 50 years after a switch (Figure 2, B; Supplementary Figure S3, A, available online). The smaller potential rebound in cervical cancer incidence compared with HPV-16 incidence is mainly due to the shift of infection to older age groups that have fewer life-years to develop cancer. The median age at HPV-16 infection is projected to increase from about age 23 years without vaccination to ages 43 and 53 years following a switch to 1-dose vaccination, assuming 25 and 35 year average duration of protection, respectively (Figure 2, C).

Because of these different key factors (ie, low HPV incidence at the time of a switch, herd effects, and shifts in the age at infection), the overall percentage of cervical cancers averted over 100 years under different pessimistic 1-dose vaccine efficacy and duration scenarios (vs no vaccination) is similar to the 2-dose scenario (about 60%) (Figure 2, D). Under the most pessimistic 1-dose scenario (vaccine duration = 25 years), 2-dose vaccination is projected to prevent 1.4 (80% uncertainty interval = 0.0-4.8) percentage points more cervical cancers over 100 years than a switch to 1-dose (Figure 2, D).

### Sensitivity analysis

**Figure 3.**
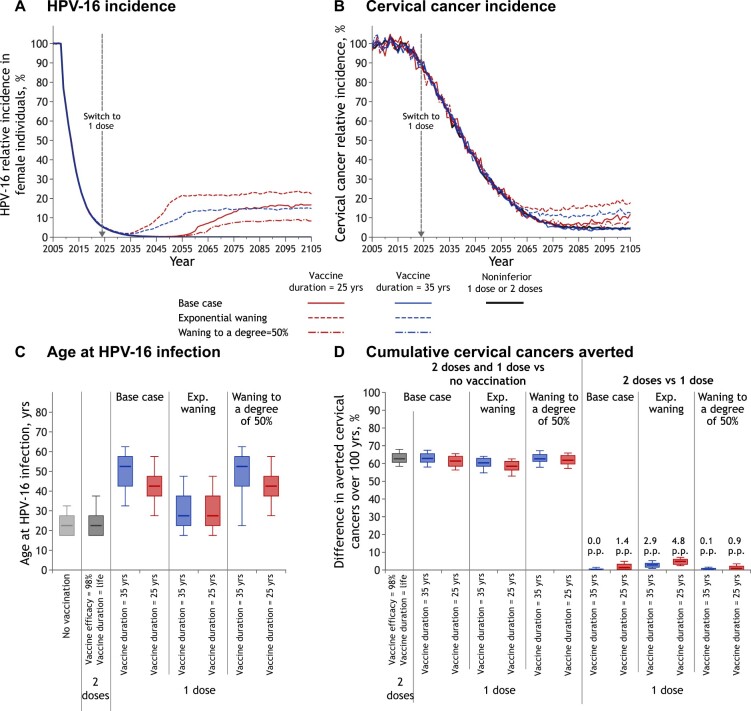
Sensitivity of 1-dose vaccination projections to mechanisms of waning protection over time. Relative incidence of **A)** HPV-16 infection among female individuals and **B)** cervical cancer (vs no vaccination). The lines overlap for all scenarios during the first years after the start of vaccination and over 100 years for the noninferior 1-dose and 2-dose scenarios and vaccine duration = 35 years scenarios with normal distributions (base case and waning to 50% degree). **C)** Age of HPV-16 acquisition among female individuals before vaccination (no vaccination) and at postvaccination equilibrium. Box plots represent the median, 10^th^, 25^th^, 75^th^, and 90^th^ percentiles of age at HPV-16 infection among the parameter sets that do not lead to elimination of HPV-16. **D)** Percentage change in the cumulative incidence of cervical cancer over 100 years (2-dose or 1-dose vaccination vs no vaccination and 2-dose vs 1-dose vaccination for different 1-dose scenarios). Box plots represent the median, 10^th^, 25^th^, 75^th^, and 90^th^ percentiles of model projections using 100 parameter sets. **Base case waning scenario**: Duration is normally distributed, and vaccinated individuals have 0% protection once they have loss of protection. **Waning to 50% degree of protection scenario**: Duration is normally distributed, and vaccinated individuals have 50% protection per sex act once they have loss of protection. **Exponential waning scenario**: Protection starts to decline immediately after vaccination, with an exponential decay, and have 0% protection after loss of protection. HPV = human papillomavirus; p.p. = percentage point difference.

**Figure 4.**
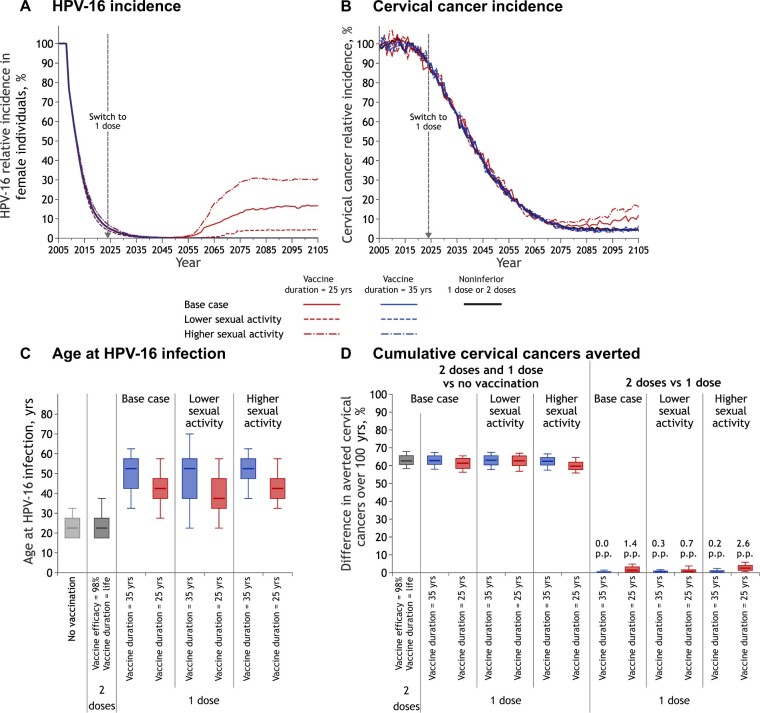
Sensitivity of 1-dose vaccination projections to sexual activity among midadults. Relative incidence of **A)** HPV-16 infection among female individuals and **B)** cervical cancer (vs no vaccination). The lines overlap for all scenarios during the first years after the start of vaccination and over 100 years for all scenarios, except vaccine duration = 25 years. **C)** Age of HPV-16 acquisition among female individuals before vaccination (no vaccination) and at postvaccination equilibrium. Box plots represent the median, 10^th^, 25^th^, 75^th^, and 90^th^ percentiles of age at HPV-16 infection among the parameter sets that do not lead to elimination of HPV-16. **D)** Percentage change in the cumulative incidence of cervical cancer over 100 years (2-dose or 1-dose vaccination vs no vaccination and 2-dose vs 1-dose vaccination for different 1-dose scenarios). Box plots represent the median, 10^th^, 25^t^^h^, 75^th^, and 90^th^ percentiles of model projections using 100 parameter sets. **Base case scenario**: Results with the 100 best fitting parameter sets. **Lower and higher sexual activity scenarios**: Base case partner separation and acquisition rates among individuals older than 30 years of age are multiplied by 0.5 and 1.5, respectively. HPV = human papillomavirus; p.p. = percentage point difference.

**Figure 5.**
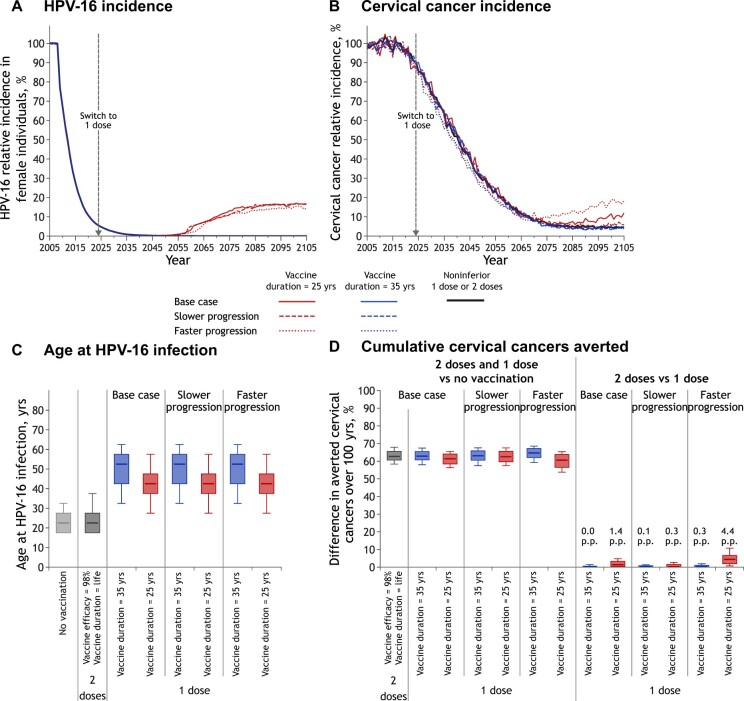
Sensitivity of 1-dose vaccination projections to progression to cervical cancer among midadults. Relative incidence of **A)** HPV-16 infection among female individuals and **B)** cervical cancer (vs no vaccination). The lines overlap for all scenarios during the first years after the start of vaccination and over 100 years for all scenarios, except vaccine duration = 25 years. **C)** Age of HPV-16 acquisition among female individuals before vaccination (no vaccination) and at postvaccination equilibrium. Box plots represent the median, 10^th^, 25^th^, 75^th^, and 90^th^ percentiles of age at HPV-16 infection among the parameter sets that do not lead to elimination of HPV-16. **D)** Percentage change in the cumulative incidence of cervical cancer over 100 years (2-dose or 1-dose vaccination vs no vaccination and 2-dose vs 1-dose vaccination for different 1-dose scenarios). Box plots represent the median, 10^th^, 25^th^, 75^th^, and 90^th^ percentiles of model projections using 100 parameter sets. **Base-case scenario**: Results with the 100 best fitting parameter sets. **Slower and faster progression scenarios**: Base case progression rates from incident infection to cervical cancer among individuals older than 30 years of age are multiplied by 0.5 and 2. HPV = human papillomavirus; p.p. = percentage point difference.

Model projections of the population-level impact of switching to 1-dose vaccination were most sensitive to assumptions about the mechanism of waning of vaccine protection, sexual activity, and progression to cervical cancer among midadults (Figures 3-5; Supplementary Figure S3, available online).

If waning of protection begins immediately after vaccination (with an exponential function) rather than protection remaining stable before declining (base case = normal distribution), rebounds in HPV infection and cervical cancer are projected to be greater and occur earlier. For the same pessimistic average duration of protection of 25 years, exponential waning of protection is projected to lead to rebounds in HPV-16 and cervical cancer incidence that are 20 years earlier than with a normal distribution (Figure 3). Furthermore, assuming an average duration of protection of 35 years, exponential waning leads to rebounds in HPV infection and cervical cancer incidence, whereas a similar average duration with a normal distribution does not. The key factor driving these differences in projections is the level of protection during peak ages of sexual activity. With an exponential function, younger, more sexually active adults can be infected (eg, >30% of individuals would be susceptible 10 years after vaccination, assuming vaccine duration = 25 years) (Figure 1), leading to a younger age at infection and less direct protection and herd effects from vaccination (Figure 3, C). In contrast, with the base case normal distribution of protection scenario, which reproduces the high sustained level of immunity seen in studies for 12 to 16 years, younger adults remain protected. Finally, even under the most pessimistic exponential waning function (vaccine duration = 25 years), a switch to 1 dose is projected to prevent a similar percentage of cervical cancers over 100 years as 2-dose vaccination (Figure 3, D).

Assumptions about the average number of new sex partners or progression from infection to cervical cancer among midadults (ie, after age 35 years) can mitigate or exacerbate the population-level impact of the limited duration of 1-dose protection (Figures 4 and 5). When assuming an average duration of protection of 25 years, the projected rebound in cervical cancer increases substantially, from about 5 percentage points at equilibrium to 10 to 15 percentage points when artificially inflating the rate of new sexual partners 1.5-fold and progression to cervical cancer 2-fold compared with base case estimates from model calibration (Figures 4, B and 5, B). In contrast, if the rate of new sexual partners is lower among midadults or if progression is slower, the potential for rebounds in HPV infection or cervical cancer incidence are substantially reduced.

Finally, model results were robust to variations in vaccination coverage and vaccination programs as well as the model used (Supplementary Figures S4-S9, available online). When using pessimistic assumptions about duration of protection (vaccine duration = 25 years), however, projected rebounds in HPV infection and cervical cancer incidence following a switch to 1-dose vaccination were slightly higher under scenarios with lower population vaccination coverage (Supplementary Figures S4-S8, available online) because they produce smaller herd effects to unprotected individuals. Even under the most pessimistic assumptions of 1-dose duration of protection (vaccine duration = 25 years), gender-neutral 1-dose vaccination with 85% coverage has an equal or higher number of cervical cancers prevented than 2-dose girls-only vaccination with 65% to 85% coverage or gender-neutral vaccination with 65% coverage (Supplementary Figure S7, D and F, available online), illustrating that the greatest projected population-level impact is achieved by vaccinating as many individuals with at least 1 dose as possible (vs 2 doses with lower coverage).

## Discussion

In high-income countries with high HPV vaccination coverage, 1-dose vaccination is projected to avert similar numbers of cervical cancers as 2 doses. Even under the more pessimistic assumption of vaccine duration (vaccine duration = 25 years), a switch to 1-dose vaccination is projected to lead to a limited rebound in HPV infection and cervical cancer incidence because 1) switching to 1-dose vaccination would occur when HPV prevalence is low due to high 2-dose vaccination coverage in high-income countries and 2) individuals would be protected during their peak ages of sexual activity, providing herd effects to unprotected adults. Furthermore, if vaccine protection wanes substantially in the long term, older adults have fewer new sexual partners, reducing their risk of infection and, if infected, have fewer life-years left to progress to cervical cancer. Projections were robust using different vaccine efficacy mechanisms and vaccination program characteristics and across different models used for projections.

### HPV vaccination policy implications

Our findings have important implications for HPV policy decisions in high-income countries because many countries are examining whether to switch to 1-dose vaccination. A main source of concern, which has led some high-income countries to continue with 2 doses, is that 1-dose vaccine efficacy and duration of protection could be inferior to 2 doses and jeopardize the gains observed from HPV vaccination over the past decade. Our model-based results suggest that 1-dose vaccination is unlikely to produce a substantial rebound in HPV infection and cervical cancer incidence and that the second dose provides little incremental benefit in terms of cervical cancer prevention at the population level. Hence, similar to modeling results for LMICs (5,6), our results suggest that providing the second dose would be an inefficient use of HPV vaccines (and that 1 dose would be highly efficient). Furthermore, under the more pessimistic assumptions of vaccine efficacy, duration of protection, and coverage, 1-dose vaccination is projected to reduce cervical cancer incidence by more than 70% in the long term (ie, at equilibrium), which would lead to cervical cancer elimination in most high-income countries in North America, Europe, and Australia (eg, average age-standardized incidence in high-income countries is currently about 7.5/100 000 women-years, elimination threshold = 4/100 000 women-years) (64,65). Our findings also suggest that if waning occurs faster than anticipated (eg, most pessimistic assumption, vaccine duration = 25 years), a rebound in HPV-16 infection would slowly start around 2050. Assuming a 25 year average duration of protection, the CVT, which started in 2005 (3,8), would be able to detect waning before 2030-2031, providing high-income countries up to 20 years to introduce mitigation strategies. We have previously shown that switching to a 2-dose strategy (with or without catch-up) is projected to mitigate any potential losses in cancer prevention (5).

There remain important inequalities in vaccination coverage, cervical cancer screening, and cervical cancer burden between population subgroups (eg, race and ethnicity, urban vs rural, deprivation scores) (41,66-73). The COVID-19 pandemic has also transiently reduced vaccination coverage in high-income countries, mainly among vulnerable population subgroups (74-76). Lower vaccination coverage among underscreened and high-burden subgroups and regions could reduce the population-level impact of HPV vaccination and increase health inequalities (66,77). The economic savings by switching to 1-dose vaccination and its programmatic flexibility could allow high-income countries to invest in increasing vaccination coverage, particularly among high-burden subgroups, to reduce inequalities and accelerate cervical cancer elimination. Future work could explore the optimal use of existing vaccine supply in high-income countries to close the gap between population subgroups.

Finally, our model projects that even under the most pessimistic assumptions of 1-dose duration of protection, gender-neutral 1-dose vaccination with high coverage prevents more cervical cancers than 2-dose vaccination with lower girls-only or gender-neutral vaccination coverage, illustrating that the greatest population-level impact is achieved by vaccinating as many individuals as possible, especially before sexual debut, with at least 1 dose.

### Research implications and value for information

Our results highlight the key factors that can have the least and greatest impact on the potential population-level effectiveness of 1-dose vaccination in high-income countries, which can help identify research priorities with the greatest value to inform policy decisions. Differences between 1-dose and 2-dose vaccine efficacy had no impact on 1-dose population-level projections under the scenarios investigated. The KEN SHE trial has shown 98% 1-dose vaccine efficacy against persistent HPV-16/18 infection, while effectiveness studies from India and Thailand have shown vaccine efficacy above 90% (9,10,78). Our results show that herd effects from high HPV vaccination coverage would mitigate any differences in vaccine efficacy between 1 and 2 doses, even under a pessimistic assumption of 90%. Previous modeling in LMICs has produced similar conclusions, with vaccine efficacy as low as 85% (5,6,79). Greater uncertainty remains about 1-dose vaccine efficacy among boys because of a lack of trial data. Our model projects, however, that herd effects from gender-neutral vaccination would mitigate the population-level impact of low 1-dose vaccine efficacy for boys (vaccine efficacy = 70% for boys). These model results, along with current available empirical evidence, can be used to evaluate the added value of or need for further large 1-dose vaccine efficacy trials in female and male individuals to make policy decisions. Additional research with the greatest value for 1-dose vaccination decisions is related to the duration of protection. Our results suggest that 1-dose duration of protection must remain high and stable during the peak ages of sexual activity. Currently, after more than 12 and 16 years of follow-up, there is no indication of significant waning of vaccine protection in the IARC India study and CVT, respectively (8,10). It is important to continue these long-term studies and to conduct population-based surveillance studies of HPV prevalence over time, particularly in countries that switch to 1-dose vaccination.

### Limitations and Strengths

The findings of our modeling study should be interpreted in light of its limitations. First, we explored several uncertainties related to the data used for model projections and highlighted the impact by performing extensive sensitivity analyses. Currently, there are limited data to inform the function of duration of protection for 1 or 2 doses, except that exponential waning is highly unlikely as this would already have been seen in long-term follow-up of the CVT and IARC India study (3,8-10). Given this, we performed extensive sensitivity analysis of duration of protection, from noninferior to pessimistic scenarios. The most pessimistic scenario of waning efficacy (average vaccine duration = 25 years) assumes a drop in protection 15 years after vaccination to reproduce an unlikely situation where the CVT trial would start showing steep declines in antibodies after the current follow-up of 16 years (8). There are also limited data on the number of new sexual partners and progression to cervical cancer among midadults. Available population-based data on the number of lifetime partners suggest that the average number of new sexual partners after age 35 years is relatively low compared with adults aged 20 to 35 years of age (46-49). Our model slightly overestimated data on the lifetime number of partners, particularly for women older than 40 years of age (Supplementary Figure S1, available online) and therefore may overestimate the potential rebound in HPV and cervical cancer incidence under pessimistic scenarios of 1-dose duration of protection or could reproduce a situation where the number of new sexual partners increases in the future amongst midadults. Finally, there are conflicting results regarding the impact of infection acquisition at an older age on progression to high-grade lesions (and thus cervical cancer), with some studies finding no impact of age on risk of progression of “newly” detected infection (50,51), while others have found a lower or higher risk of progression (52). We varied the above key parameters over wide ranges; therefore, model conclusions and insights are robust to these main sources of uncertainty. Nevertheless, more research is needed among midadults to enable more precise projections of the population-level impact of HPV vaccination if 1-dose or 2-dose vaccination programs start to show waning of protection. It is important to note that the model projections in this study are not meant to be interpreted as predictions because they do not account for unpredictable changes in behavior or policy (apart from those modeled) that may occur in the decades to come. Rather, they are meant to illustrate and explain how different factors (such as vaccine efficacy and duration) can affect the population-level effectiveness of 1-dose vaccination in different high-income country settings.

Our modeling analysis has also several strengths. First, HPV-ADVISE projections were done using models calibrated to 2 countries (the United States and Canada), considering uncertainty around sexual behavior and biological parameters as well as variability in the epidemiological data. Furthermore, between-model validation was performed using the Harvard model showing similar findings, and extensive sensitivity analysis was performed. Our main conclusions are likely generalizable to various high-income country settings in Europe, North America, and Australia/New Zealand, given that we have used multiple models that integrate different sexual behavior and HPV epidemiological profiles and show robustness of conclusions for both girls-only and gender-neutral vaccination using high (85%) and medium (65%) vaccination coverage from countries within these regions. One must be careful, however, when generalizing projections to high-income countries in other regions, such as east Asia or Eastern Europe, that have different sexual behavior or HPV and cervical cancer epidemiology (80-82).

In conclusion, if vaccine protection remains high during the peak ages of sexual activity, 1-dose vaccination is projected to avert similar numbers of cervical cancers as 2 doses in high-income countries. Under the most pessimistic assumptions of duration of protection, a limited rebound in HPV infection and cervical cancer incidence is projected to occur 25 years and 50 years, respectively, after a switch from 2 doses. Hence, if 1-dose duration of protection is shorter than anticipated, data from the IARC India study and CVT, which have already accrued about 15 years of follow-up data, would show waning protection before a potential increase in HPV infection or cervical cancer incidence in high-income countries, leaving time to decide whether to introduce mitigation strategies.

## Supplementary Material

lgae038_Supplementary_Data

## Data Availability

Model outputs are available upon reasonable request.
