# Supplementary material for: Population-level impact of switching to 1-dose human papillomavirus vaccination in high-income countries: examining uncertainties using mathematical modeling
Source: J Natl Cancer Inst Monogr. 2024 Nov 12;2024(67):387–99. doi: 10.1093/jncimonographs/lgae038 (PMC11555275; doi:10.1093/jncimonographs/lgae038)
Supplement: lgae038_Supplementary_Data [file lgae038_supplementary_data.pdf]

SUPPLEMENTARY MATERIAL

Population-level impact of switching to one-dose HPV vaccination in high-income countries: Examining uncertainties using mathematical modeling

Marc Brisson, Jean-François Laprise, Mélanie Drolet, Éléonore Chamberland, Élodie Bénard, Emily Burger, Mark Jit, Jane Kim, Chantal Sauvageau, Stephen Sy

CONTENT

Table S1. HPV Frame

Figure S1. Median number of lifetime partners

Figure S2. Projected population-level impact of switching to one-dose HPV vaccination for different vaccine efficacy and vaccine duration of protection scenarios

Figure S3. Difference in cervical cancer incidence at equilibrium (two- or one-dose vaccination versus no vaccination and two-dose versus one-dose vaccination for different one-dose scenarios)

Figure S4. Assuming girls-only vaccination with 85% coverage, projected population-level impact of switching to one-dose HPV vaccination for different vaccine efficacy and vaccine duration of protection scenarios

Figure S5. Assuming gender-neutral vaccination with 65% coverage, projected population-level impact of switching to one-dose HPV vaccination for different vaccine efficacy and vaccine duration of protection scenarios

Figure S6. Assuming girls-only vaccination with 65% coverage, projected population-level impact of switching to one-dose HPV vaccination for different vaccine efficacy and vaccine duration of protection scenarios

Figure S7. Assuming different vaccination programs and coverage, model projections of the relative incidence of HPV-16 infection among females (vs no vaccination)

Figure S8. Assuming different vaccination programs and coverage and one-dose vaccine efficacy and duration of protection, model projections of the percent change in the cumulative incidence of cervical cancer over 100 years (versus no vaccination)

Figure S9. Between-model validation. Comparison of the HPV-ADVISE and Harvard model projections of the population-level impact of switching to one-dose vaccination for different vaccine duration of protection scenarios.

Table S1. HPV Frame[1]

A. Inputs	Reported by age? (Y/N)	Reported by sex? (Y/N)	Comments
**Core reporting standard**			
Target population for intervention	Y	Y	Vaccination of 10-year-old girls with a 11-19-year-old catch-up and of 10-year-old boys with a 11-14-year-old catch-up (see section Methods of the article).
Sexual behavior	Y	Y	Sexual behavior inputs used (number of lifetime partners, number of partners in the last year, partnership formation, etc) are described in the Technical Appendix*, section 2.2.2
Cohort examined for evaluation/ time horizon	Y (multiple cohorts)	Y (multiple cohorts)	100-year time horizon from start of vaccination (2008-2108). Intervention is given to cohorts, but we examine the outcome in the population.
Quality of life assumptions	Not applicable	Not applicable	This study focuses on the impact of vaccination on health outcomes only.
Calibration	Y	Y	HPV-ADVISE HIC was calibrated with country-specific behavioral and epidemiological data (see Technical Appendix*, section 2.3).
Validation (where possible)	Y	Y	HPV-ADVISE HIC has been validated and previously used to model various HPV vaccination/cervical screening strategies (see technical Appendix, section 2.4). We have performed model validation compared to post-vaccination empirical data[2,3]. and comparative model validation with other independent models (projections were consistent between models[4-6]).
Costs	Not applicable	Not applicable	This study focuses on the impact of vaccination on health outcomes only.
**Reporting standards for models of vaccination in adolescent individuals**			
Vaccine coverage	Y	Y	Intervention is for girls and boys. See section Methods of the article for specific coverage.
Vaccine efficacy	Y	Y	See section Methods of the article for specific efficacy.
Vaccine cross-protection	Not applicable	Not applicable	The study focuses on the nonavalent vaccine, which includes oncogenic types HPV16, 18, 31, 33, 45, 52 and 58.
Duration vaccine protection and waning	Y	Y	See section Methods of the article for assumptions on duration of protection.
Vaccine and delivery costs	Not applicable	Not applicable	This study focuses on the impact of vaccination on health outcomes only.
Pre-vaccination disease burden	Y	Y	Pre-vaccination epidemiological data were used for calibration (see Technical Appendix*, section 2.3).
Duration of natural immunity	Y	Y	See Technical Appendix*, section 2.2.3

A. Inputs	Reported by age? (Y/N)	Reported by sex? (Y/N)	Comments
**Reporting standards for evaluations assessing alternative vaccine types or reduced-dose schedules**			
Timing between doses	Y	Y	Not applicable for one-dose schedule. For two doses, we modeled HPV vaccination program representing HICs programs and 2-doses recommendations (i.e. 6 months between doses). See section Methods of the article.
**Reporting standards for models of HPV prevention in LMIC**			
HIV prevalence rates, if endemic in country	Not applicable	Not applicable	HIC only
Description of any opportunistic or pilot/demonstration screening projects	Not applicable	Not applicable	HIC only

B. Outputs	Reported by age? (Y/N)	Report by sex? (Y/N)	Comments
**Core reporting standards**			
Cancer incidence, mortality, life years, QALYs/DALYs (as appropriate)	Y	Y	Our main outcomes were the change in cervical cancer incidence, the cumulated number of cervical cancers averted and the change in age at HPV-16 infection among females (see section Methods and Results of the article). We also report change in incidence of HPV-16 and HR-HPV infection among females and males (Figure S2).
HPV prevalence, pre-intervention	Y	Y	Pre-intervention prevalence is used for calibration and is reported in the Technical Appendix*, section 2.3.
CIN2/3 detected	N	N	This outcome is not reported as the present study focuses on the impact of vaccination on cervical cancer.
Sensitivity analysis on key inputs	Y	Y	Sensitivity analyses were performed. See sections Methods and Results of the article.
Incremental cost-effectiveness ratios and costs saved	Not applicable	Not applicable	This study focuses on the impact of vaccination on health outcomes only.
**Reporting standards for models of vaccination in adolescent individuals**			
Absolute reductions in HPV infections, and/or warts, post-vaccination	Y	Y	We present the relative change in HPV-16 infection incidence among females (vs no vaccination) and the average age of HPV-16 infection among females. For some scenarios, we present the relative change in HPV-16 infection incidence among males and the relative change in HR-HPV infection incidence among males and females (Figure S2).
Absolute reductions in CIN2+ post-vaccination	N	N	Absolute reductions in CIN2+ are not presented since this study focuses on the impact of vaccination on cervical cancer.
Absolute reductions in invasive cancer (cervical and other HPV cancers, as relevant) post-vaccination	See comment	Y	We present the relative change in cervical cancer incidence, the percent change in the cumulative incidence of cervical cancer over a 100-year time horizon, and the difference in cervical cancer incidence at equilibrium (see section Methods and Results of the article).

* Technical Appendix: http://www.marc-brisson.net/HPVadvise.pdf http://www.marc-brisson.net/HPVadvise-US.pdf

1. Canfell K, Kim JJ, Kulasingam S, et al. HPV-FRAME: A consensus statement and quality framework for modelled evaluations of HPV-related cancer control. *Papillomavirus Res*. 2019;8:100184.
2. Laprise J-F, Chesson HW, Markowitz LE*, et al.* Effectiveness and Cost-Effectiveness of Human Papillomavirus Vaccination Through Age 45 Years in the United States. Annals of Internal Medicine 2020;172(1):22-29.
3. Drolet M, Laprise J-F, Brotherton JML*, et al.* The Impact of Human Papillomavirus Catch-Up Vaccination in Australia: Implications for Introduction of Multiple Age Cohort Vaccination and Postvaccination Data Interpretation. Journal of Infectious Diseases 2017;216(10):1205-1209.
4. Brisson M, Bénard É, Drolet M*, et al.* Population-level impact, herd immunity, and elimination after human papillomavirus vaccination: a systematic review and meta-analysis of predictions from transmission-dynamic models. The Lancet Public Health 2016;1(1):e8-e17
5. Brisson M, Kim JJ, Canfell K, et al. Impact of HPV vaccination and cervical screening on cervical cancer elimination: a comparative modelling analysis in 78 low-income and lower-middle-income countries. *Lancet* 2020; 395(10224): 575-90.
6. Canfell K, Kim JJ, Brisson M, et al. Mortality impact of achieving WHO cervical cancer elimination targets: a comparative modelling analysis in 78 low-income and lower-middle-income countries. *Lancet* 2020; 395(10224): 591-603.

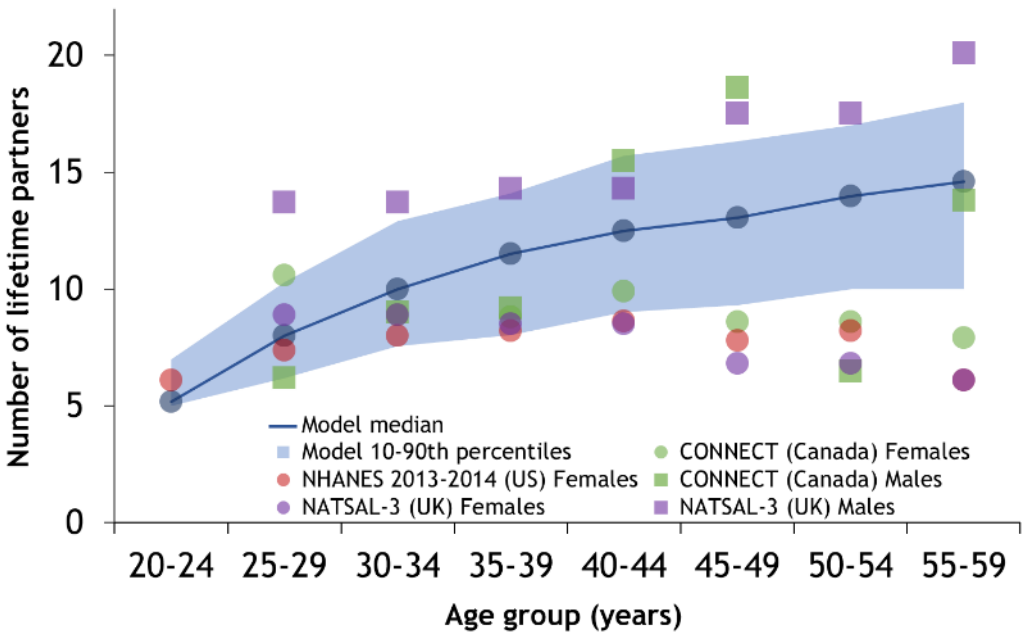

Figure S1. Median number of lifetime partners. The blue circles and shaded area represent the median and 10th to 90th percentiles range of HPV-ADVISE projections for the 100 best-fitting parameter sets representing North America for the median number of lifetime partners among females. The other circles and squares represent the observed data from different studies and locations for females and males, respectively.[1-3]

1. Haderxhanaj LT, Leichliter JS, Aral SO, Chesson HW. Sex in a lifetime: Sexual behaviors in the United States by lifetime number of sex partners, 2006-2010. Sex Transm Dis. Jun 2014;41(6):345-52. doi:10.1097/OLQ.0000000000000132.
2. Datta S, Mercer CH, Keeling MJ. Capturing sexual contact patterns in modelling the spread of sexually transmitted infections: Evidence using Natsal-3. PLoS One. 2018;13(11):e0206501. doi:10.1371/journal.pone.0206501.
3. Brisson M, Drolet M, Boily M-C. Unpublished data from CONNECT: Measuring social and sexual contact patterns in Canada to improve the control of infectious diseases. Accessed March 27, 2024. https://cirnetwork.ca/research-study/measuring-social-and-sexual-contact-patterns-in-canada-to-improve-the-control-of-infectious-diseases/

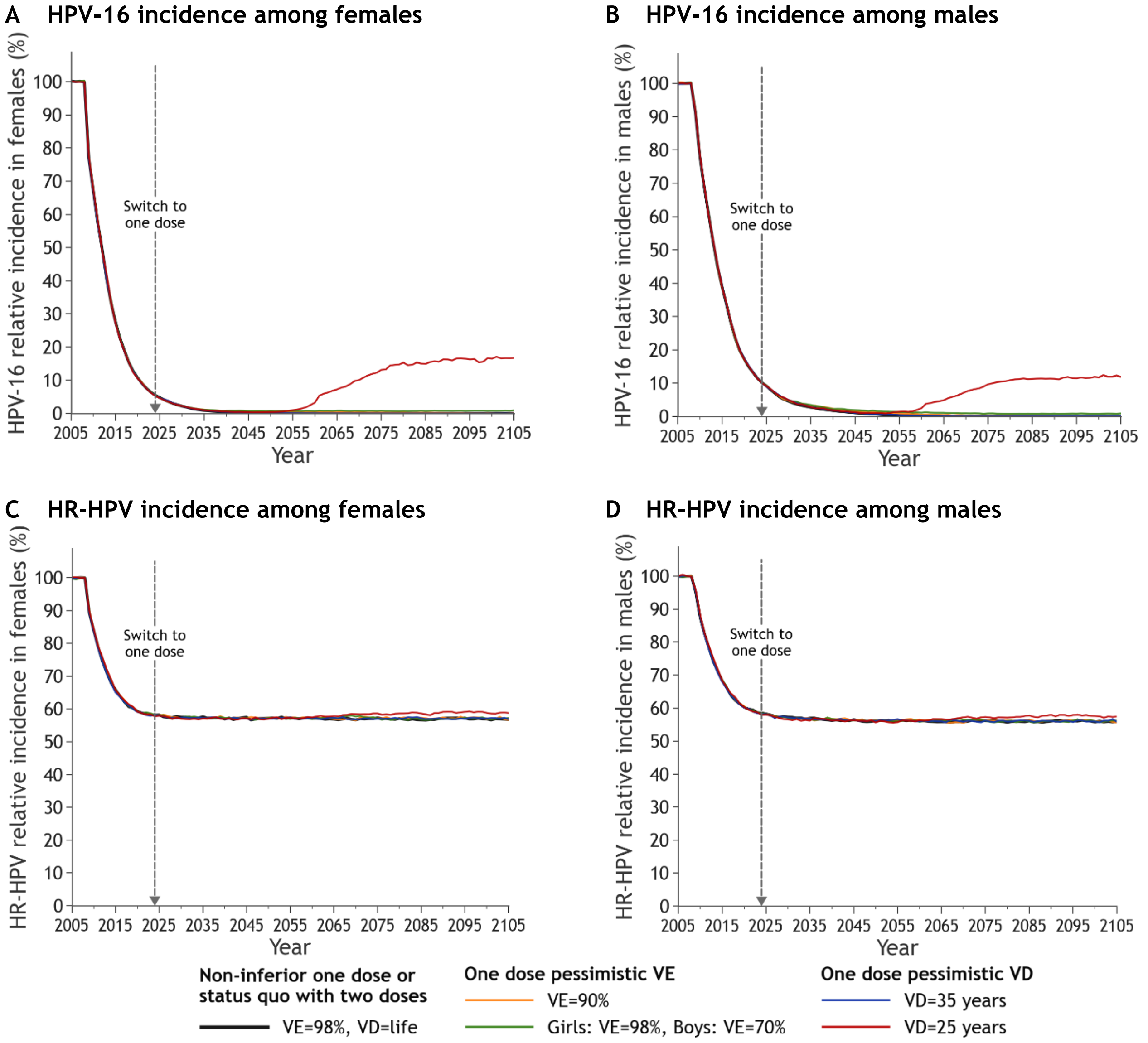

Figure S2. Projected population-level impact of switching to one-dose HPV vaccination for different vaccine efficacy and vaccine duration of protection scenarios. Relative incidence of HPV-16 infection among **A)** females and **B)** males (versus no vaccination). Relative incidence of infection with all high oncogenic risk HPV (HR-HPV) types (16, 18, 31, 33, 45, 52, 58, 35, 39, 51, 56, 59, 66, 68, 73, 82) among **C)** females and **D)** males (versus no vaccination). The lines are the median result of model projections using 100 parameter sets. The lines overlap for all scenarios during the first years after the start of vaccination, and over 100 years for all scenarios except VD=25yrs. HR-HPV=high oncogenic risk HPV; VE=vaccine efficacy; VD=vaccine duration of protection.

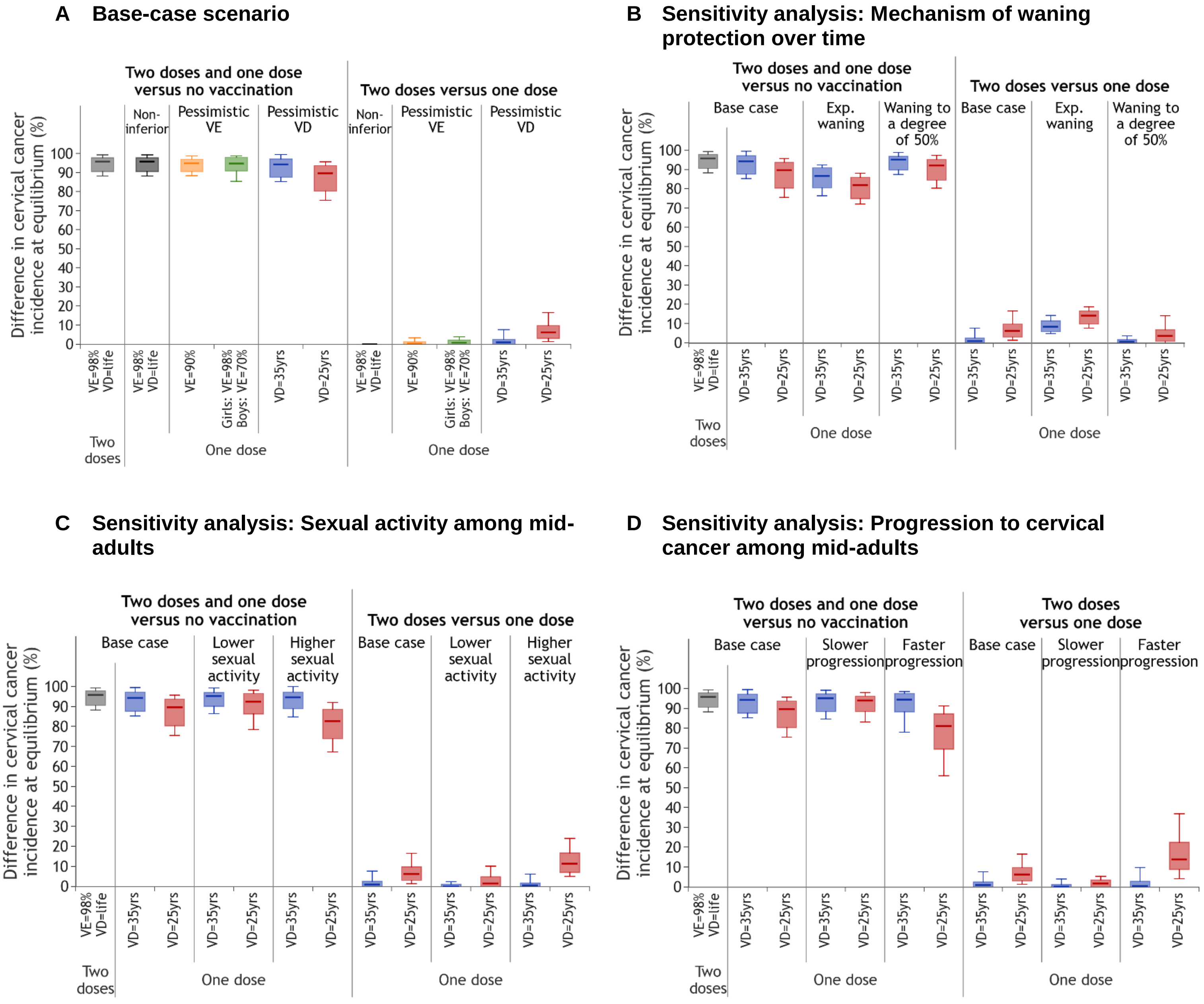

Figure S3. Difference in cervical cancer incidence at equilibrium (two- or one-dose vaccination versus no vaccination and two-dose versus one-dose vaccination for different one-dose scenarios) for **A)** Base-case scenario, **B)** Sensitivity analysis: Mechanism of waning protection over time**, C)** Sensitivity analysis: Sexual activity among mid-adults, **D)** Progression to cervical cancer among mid-adults. Boxplots represent the median, 10th, 25th, 75th and 90th percentiles of model projections using 100 parameter sets. VE=vaccine efficacy; VD=vaccine duration of protection.

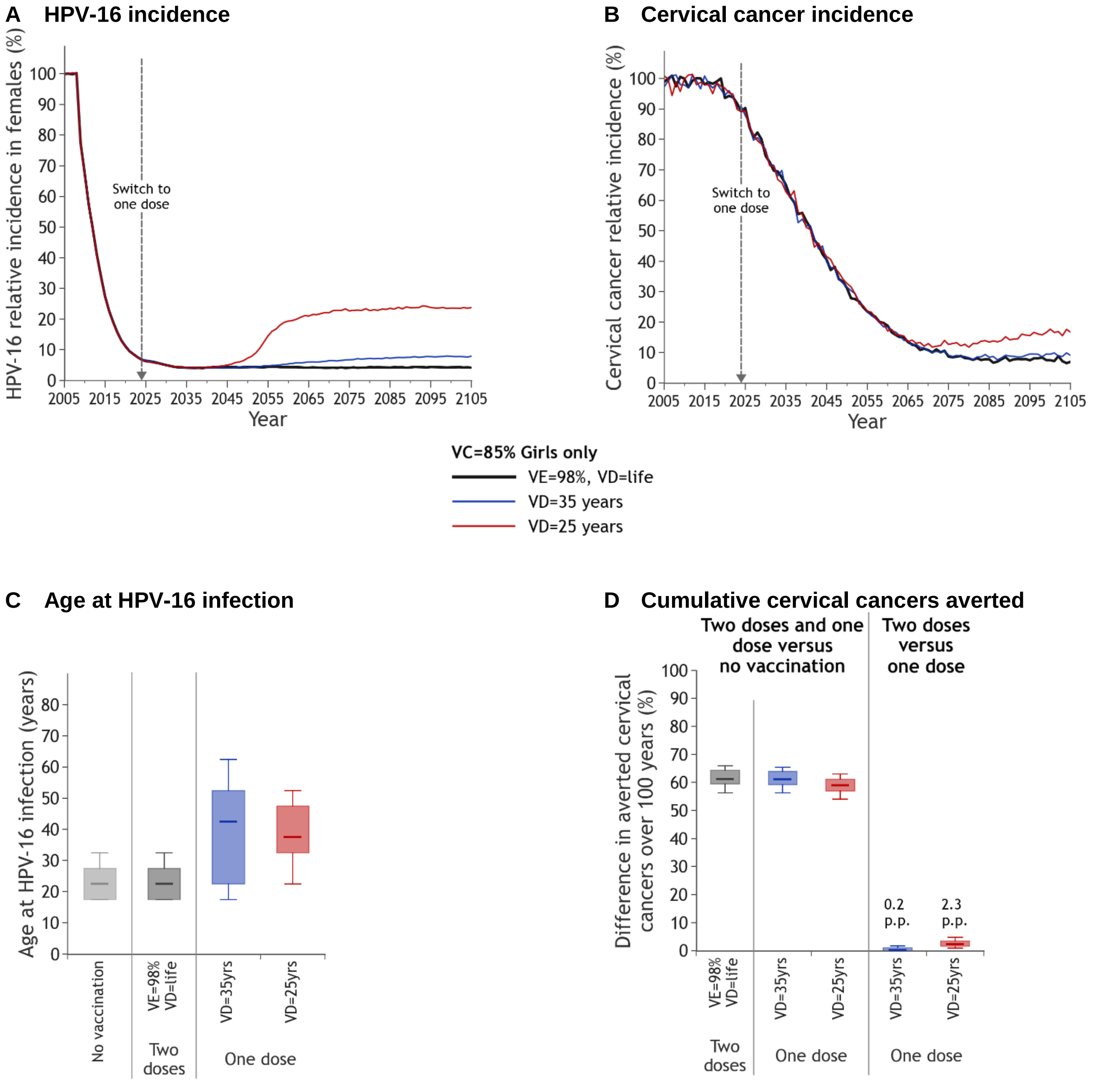

Figure S4. Assuming girls-only vaccination with 85% coverage, projected population-level impact of switching to one-dose HPV vaccination for different vaccine efficacy and vaccine duration of protection scenarios. Relative incidence of **A)** HPV-16 infection among females and **B)** cervical cancer (versus no vaccination). **C)** Age of HPV-16 acquisition among females prior to vaccination (no vaccination) and at post-vaccination equilibrium**.** Boxplots represent the median, 10th, 25th, 75th and 90th percentiles of age at HPV-16 infection among the parameter sets that do not lead to elimination of HPV-16. **D)** Percent change in the cumulative incidence of cervical cancer over 100 years (two- or one-dose vaccination versus no vaccination and two-dose versus one-dose vaccination for different one-dose scenarios)**.** Boxplots represent the median, 10th, 25th, 75th and 90th percentiles of model projections using 100 parameter sets. VC=vaccination coverage; p.p.=percentage point difference; VE=vaccine efficacy; VD=vaccine duration of protection.

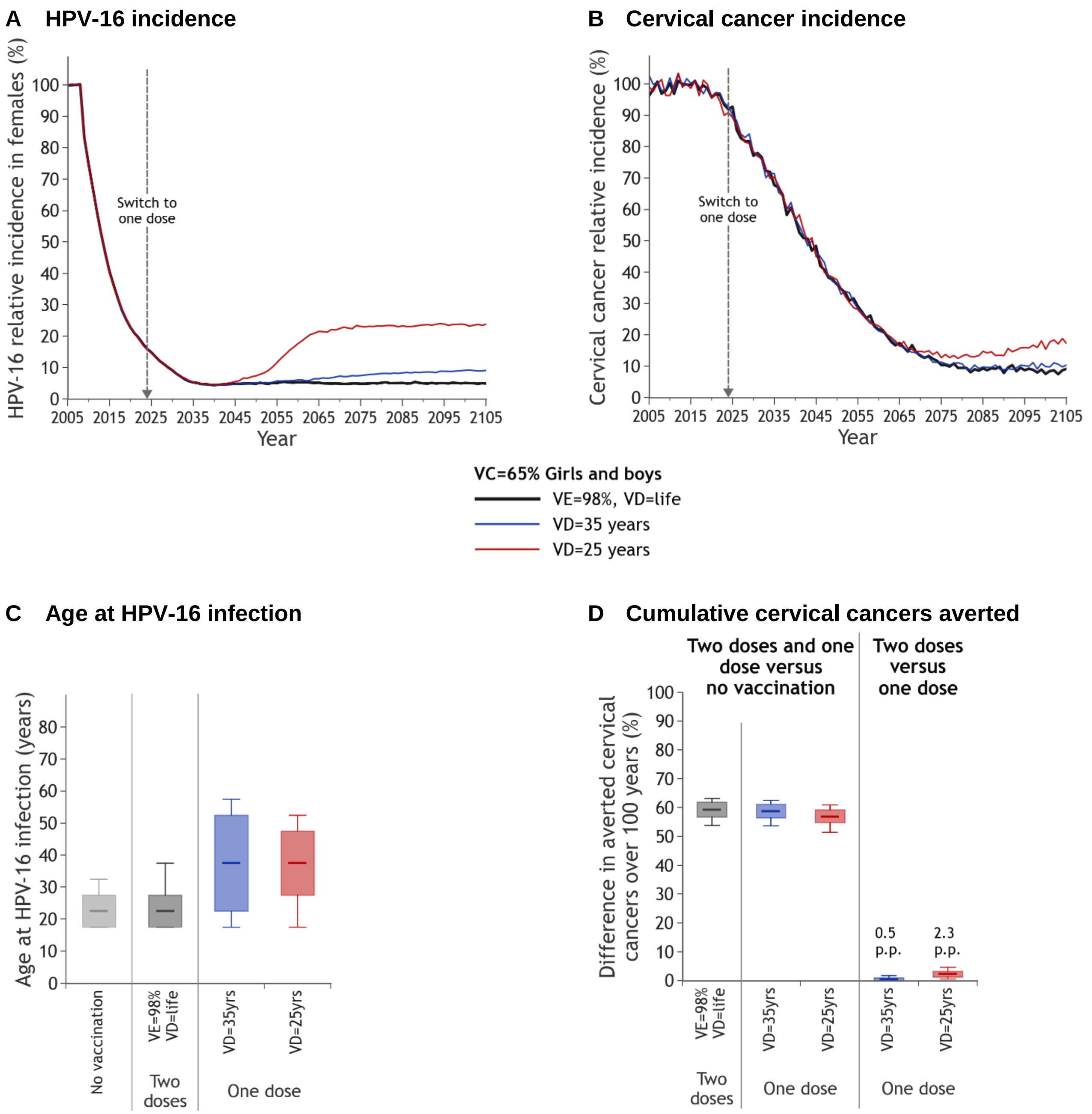

Figure S5. Assuming gender-neutral vaccination with 65% coverage, projected population-level impact of switching to one-dose HPV vaccination for different vaccine efficacy and vaccine duration of protection scenarios. Relative incidence of **A)** HPV-16 infection among females and **B)** cervical cancer (versus no vaccination). **C)** Age of HPV-16 acquisition among females prior to vaccination (no vaccination) and at post-vaccination equilibrium**.** Boxplots represent the median, 10th, 25th, 75th and 90th percentiles of age at HPV-16 infection among the parameter sets that do not lead to elimination of HPV-16. **D)** Percent change in the cumulative incidence of cervical cancer over 100 years (two- or one-dose vaccination versus no vaccination and two-dose versus one-dose vaccination for different one-dose scenarios)**.** Boxplots represent the median, 10th, 25th, 75th and 90th percentiles of model projections using 100 parameter sets. VC=vaccination coverage; p.p.=percentage point difference; VE=vaccine efficacy; VD=vaccine duration of protection.

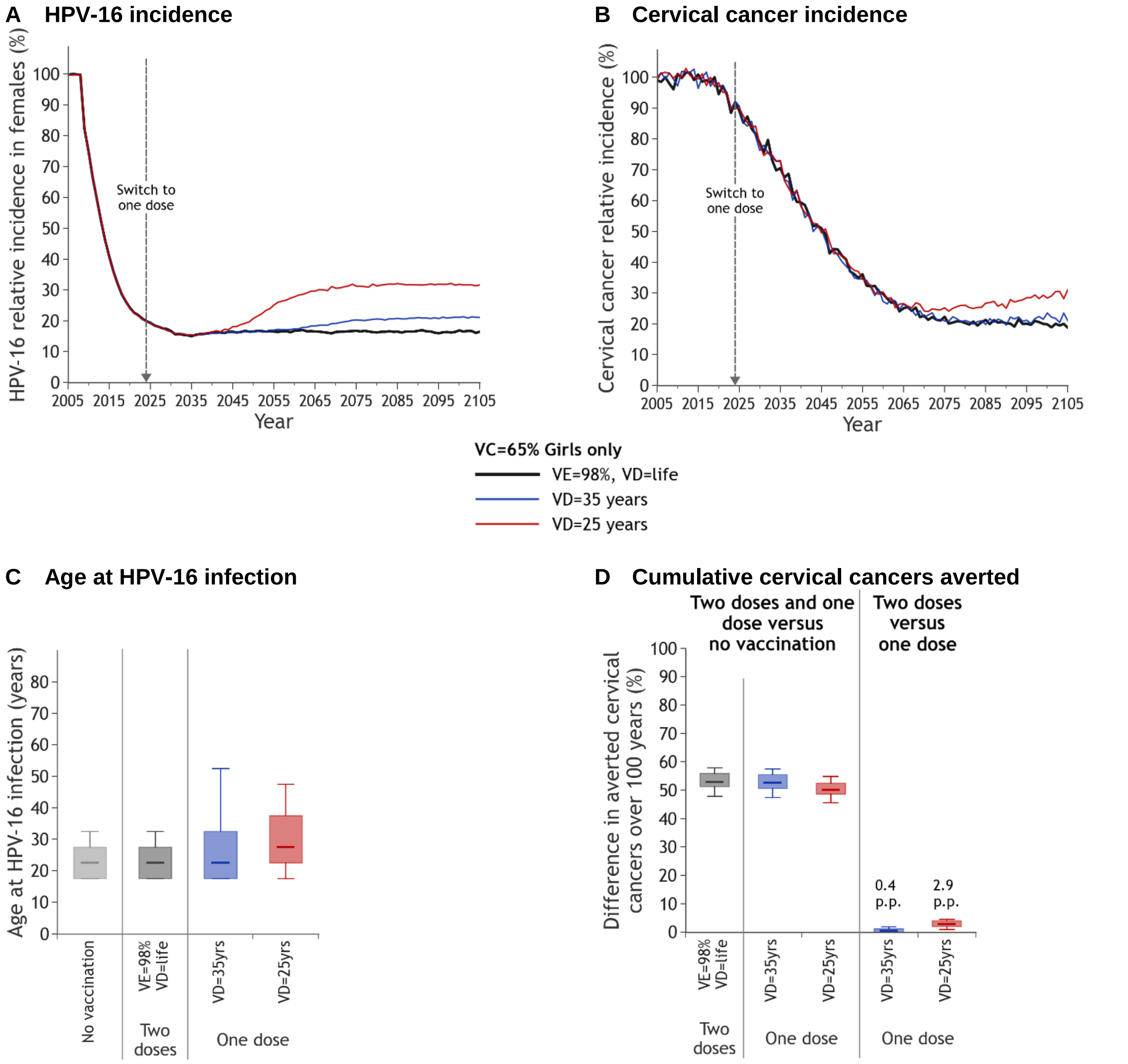

Figure S6. Assuming girls-only vaccination with 65% coverage, projected population-level impact of switching to one-dose HPV vaccination for different vaccine efficacy and vaccine duration of protection scenarios. Relative incidence of **A)** HPV-16 infection among females and **B)** cervical cancer (versus no vaccination). **C)** Age of HPV-16 acquisition among females prior to vaccination (no vaccination) and at post-vaccination equilibrium**.** Boxplots represent the median, 10th, 25th, 75th and 90th percentiles of age at HPV-16 infection among the parameter sets that do not lead to elimination of HPV-16. **D)** Percent change in the cumulative incidence of cervical cancer over 100 years (two- or one-dose vaccination versus no vaccination and two-dose versus one-dose vaccination for different one-dose scenarios)**.** Boxplots represent the median, 10th, 25th, 75th and 90th percentiles of model projections using 100 parameter sets. VC=vaccination coverage; p.p.=percentage point difference; VE=vaccine efficacy; VD=vaccine duration of protection.

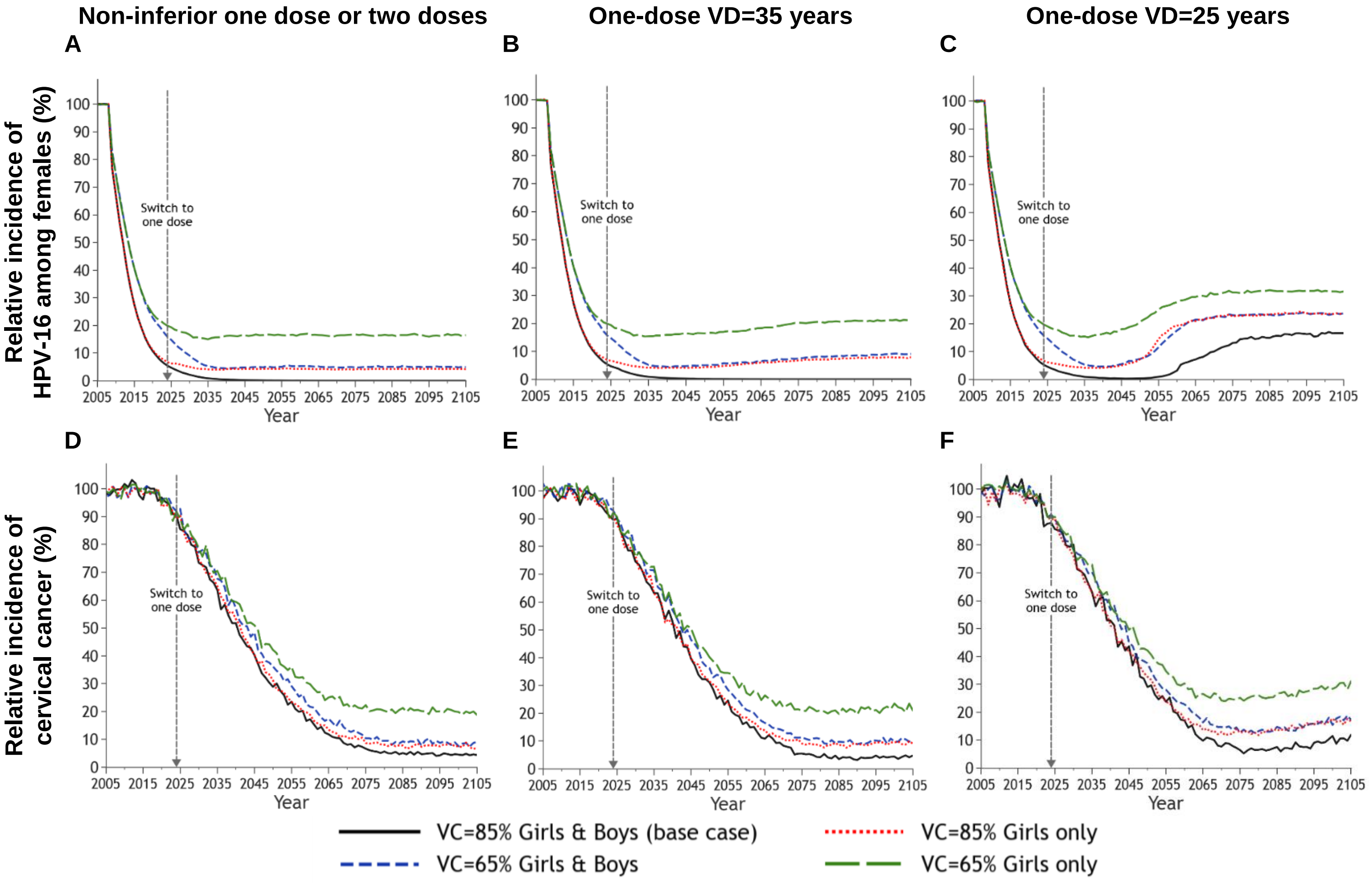

Figure S7. Assuming different vaccination programs and coverage, model projections of the relative incidence of HPV-16 infection among females (vs no vaccination) for the **A)** non-inferior one-dose or two-dose scenarios, **B)** VD=35 years scenario, and **C)** VD=25 years scenario. Relative incidence of cervical cancer (vs no vaccination) for the **D)** non-inferior one-dose or two-dose scenarios, **E)** VD=35 years scenario, and **F)** VD=25 years scenario. VC=vaccination coverage; VE=vaccine efficacy; VD=vaccine duration of protection.

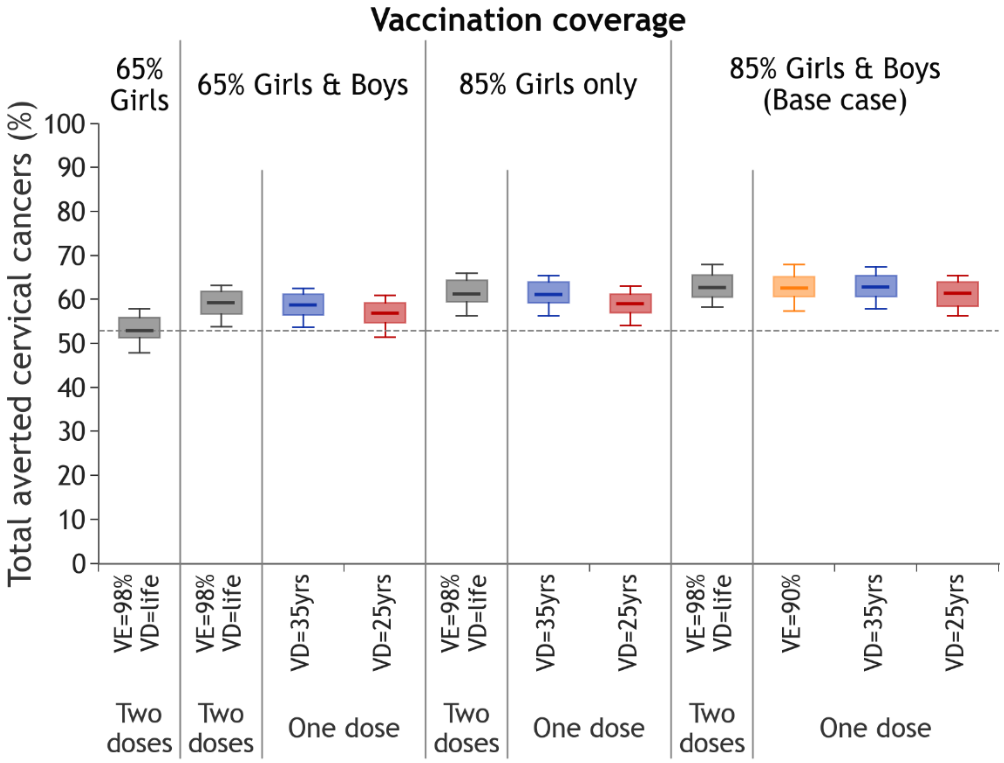

Figure S8. Assuming different vaccination programs and coverage and one-dose vaccine efficacy and duration of protection, model projections of the percent change in the cumulative incidence of cervical cancer over 100 years (versus no vaccination). Boxplots represent the median, 10th, 25th, 75th and 90th percentiles of model projections using 100 parameter sets. VE=vaccine efficacy; VD=vaccine duration of protection.

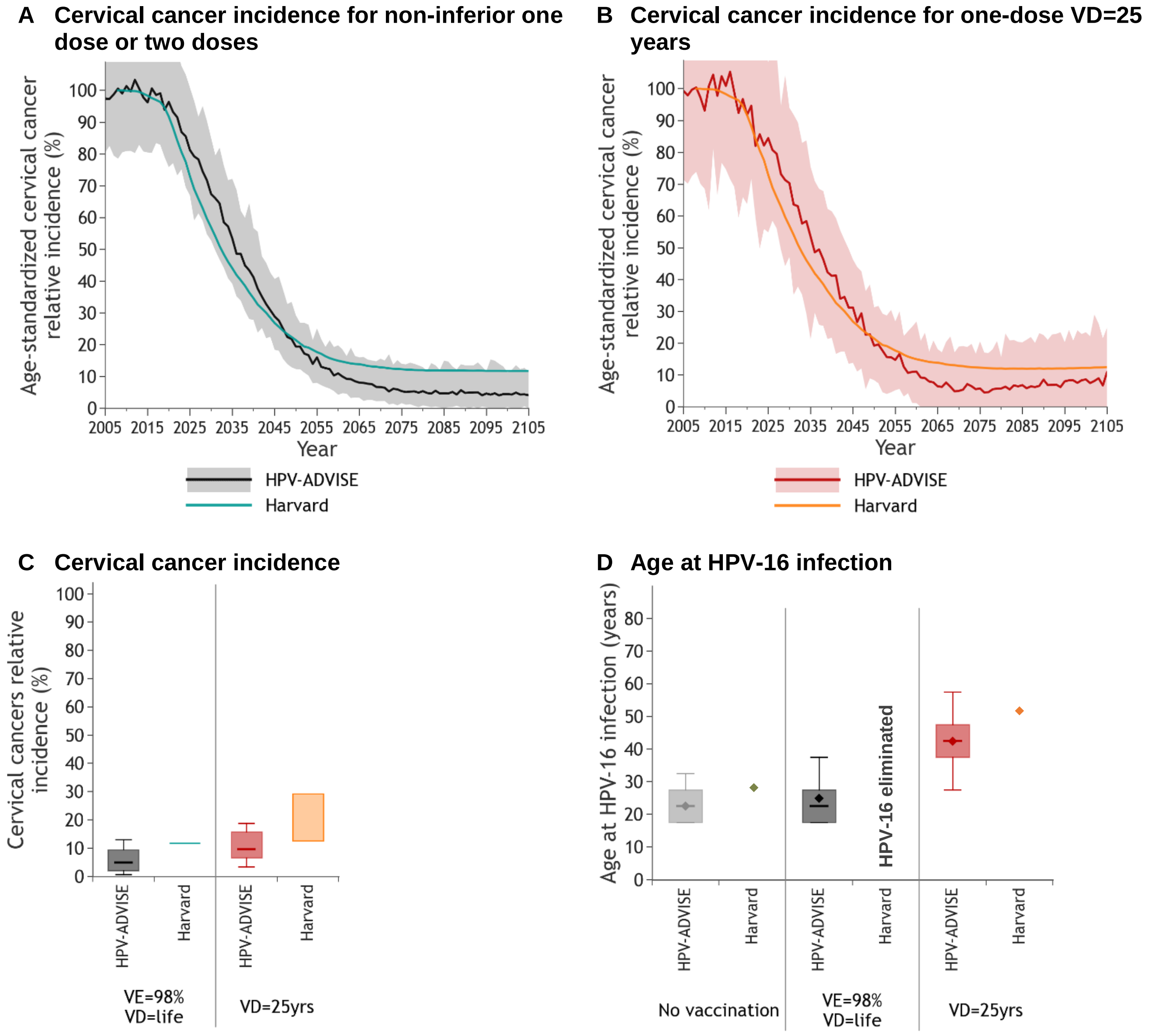

Figure S9. Between-model validation. Comparison of the HPV-ADVISE and Harvard model projections of the population-level impact of switching to one-dose vaccination for different vaccine duration of protection scenarios. Relative incidence of cervical cancer (versus no vaccination) over 100 years for **A)** the non-inferior scenario, and **B)** the most pessimistic one-dose duration of protection scenario (VD=25 years). **C)** Relative incidence of cervical cancer at post-vaccination equilibrium (versus no vaccination)**.** For panels A), B) and C) HPV-ADVISE boxplots represent the median, 10th, 25th, 75th and 90th percentiles of model projections using 100 parameter sets. Harvard box represents the range between the relative incidence after 100 years and at equilibrium. **D)** Age of HPV-16 acquisition among females prior to vaccination and at post-vaccination equilibrium**.** Diamonds and boxplots represent the mean, and median, 10th, 25th, 75th and 90th percentiles of age at HPV-16 infection among the parameter sets that do not lead to elimination of HPV-16 (for HPV-ADVISE). Incidence is age-standardized to 2015 world standard population from the United Nations World Population Prospect[1]. VE=vaccine efficacy; VD=vaccine duration of protection

1.United Nations, Department of Economic and Social Affairs, Division P. World Population Prospects: The 2017 Revision, custom data acquired via website. Accessed October 17, 2018. https://esa.un.org/unpd/wpp/dataquery
